# Opposite effects of two estrogen receptors on tau phosphorylation through disparate effects on the miR-218/PTPA pathway

**DOI:** 10.1111/acel.12366

**Published:** 2015-06-26

**Authors:** Yan-Si Xiong, Fang-Fang Liu, Dan Liu, He-Zhou Huang, Na Wei, Lu Tan, Jian-Guo Chen, Heng-Ye Man, Cheng-Xin Gong, Youming Lu, Jian-Zhi Wang, Ling-Qiang Zhu

**Affiliations:** 1 Department of Pathophysiology, School of Basic Medicine, Key Laboratory of Neurological Disorder of Education Ministry, Tongji Medical College, Huazhong University of Science and TechnologyWuhan, 430030, China; 2 The Institute for Brain Research, Collaborative Innovation Center for Brain Science, Huazhong University of Science and TechnologyWuhan, 430030, China; 3 Department of Genetics, School of Basic Medicine, Tongji Medical College, Huazhong University of Science and TechnologyWuhan, 430030, China; 4 Sino-Canada Collaborative Platform on Molecular Biology of Neurological Disease, Tongji Medical College, Huazhong University of Science and TechnologyWuhan, 430030, China; 5 Department of Pharmacology, School of Basic Medicine, Tongji Medical College, Huazhong University of Science and TechnologyWuhan, 430030, China; 6 Department of Biology, Boston UniversityBoston, MA, 02215, USA; 7 Department of Neurochemistry, Inge Grundke-Iqbal Research Floor, New York State Institute for Basic Research in Developmental DisabilitiesStaten Island, NY, 10314, USA

**Keywords:** Alzheimer’s disease, estrogen receptor, miR-218, PTPα, tauopathy

## Abstract

The two estrogen receptors (ERs), ERα and ERβ, mediate the diverse biological functions of estradiol. Opposite effects of ERα and ERβ have been found in estrogen-induced cancer cell proliferation and differentiation as well as in memory-related tasks. However, whether these opposite effects are implicated in the pathogenesis of Alzheimer’s disease (AD) remains unclear. Here, we find that ERα and ERβ play contrasting roles in regulating tau phosphorylation, which is a pathological hallmark of AD. ERα increases the expression of miR-218 to suppress the protein levels of its specific target, protein tyrosine phosphatase α (PTPα). The downregulation of PTPα results in the abnormal tyrosine hyperphosphorylation of glycogen synthase kinase-3β (resulting in activation) and protein phosphatase 2A (resulting in inactivation), the major tau kinase and phosphatase. Suppressing the increased expression of miR-218 inhibits the ERα-induced tau hyperphosphorylation as well as the PTPα decline. In contrast, ERβ inhibits tau phosphorylation by limiting miR-218 levels and restoring the miR-218 levels antagonized the attenuation of tau phosphorylation by ERβ. These data reveal for the first time opposing roles for ERα and ERβ in AD pathogenesis and suggest potential therapeutic targets for AD.

## Introduction

Alzheimer’s disease (AD), the most common form of dementia, was first reported in 1906 by Alois Alzheimer. Extensive research has established the two most prominent pathological hallmarks in AD brains: senile plaques and neurofibrillary tangles (NFTs). The degree of cognitive impairment has been shown to significantly correlate with the presence of NFTs (Braskie *et al*., 2010).

Hyperphosphorylated tau, which forms paired helical filaments, is the major component of NFTs (Johnson & Jenkins, 1999). Although the precise role of tau phosphorylation in the toxicity remains unclear, the abnormalities caused by hyperphosphorylated tau have been well studied. For example, abnormal tau hyperphosphorylation converts normal tau from a microtubule assembly-promoting to a microtubule-disrupting protein (Alonso *et al*., 1994). In AD brains, tau is hyperphosphorylated about three times more than that in normal brains, and it promotes misfolding of normal tau and coaggregates with it into filaments (Alonso *et al*., 1996). The levels of tau phosphorylation are positively correlated with cognitive deficits in multiple animal models and patients with AD (Mitchell *et al*., 2002; Zhu *et al*., 2004; Stancu *et al*., 2014). Therefore, the development of tau-based therapeutic drugs for AD-related tauopathies will require the elucidation of the underlying mechanisms of how the abnormal phosphorylation is regulated.

Previous studies have shown that the incidence of AD in women is significantly higher than that in men (Lee *et al*., 2014), and this difference has been attributed to the loss of estrogen and a variety of related mechanisms at the molecular, cellular, and hormonal levels. Subsequent studies have elucidated the neuroprotective roles of estrogen against AD-related pathology and have proposed that the beneficial effects of estrogen on AD are directly linked to its ability to reduce amyloid-β peptide and tau aggregates (Vest & Pike, 2013). There are two known estrogen receptors (ERs), usually referred to as ERα and ERβ and both widely distributed in the brain (Perez *et al*., 2003). In the brain of patients with AD, both ERα and ERβ are abnormally regulated. For example, the mitochondrial ERβ is reduced in the frontal cortex of female patients with AD (Long *et al*., 2012), and the alternative splicing of ERα mRNA is diminished in the AD brain especially in female cases (Ishunina & Swaab, 2012). Paradoxically, in the hippocampus of patients with AD, the ERα-expressing neurons are decreased (Hu *et al*., 2003), while the ERβ immunoreactivity is increased (Savaskan *et al*., 2001). As previously reported, neuroprotection against β-amyloid toxicity by estrogen administration requires the expression of ERα or ERβ, as well as activation of the mitogen-activated protein kinase pathway (Fitzpatrick *et al*., 2002).

These studies indicate the potential roles for ERα and ERβ in the pathogenesis of AD. However, the specific effect of ERα or ERβ in tauopathy is still elusive. Here, using specific human ERα and ERβ plasmids and small interfering RNA transfection, we demonstrated that ERα positively, whereas ERβ negatively, regulated the phosphorylation levels of tau protein. Interestingly, the distinct regulation of tau phosphorylation by ERα and ERβ resulted from their opposite regulatory role on miR-218, resulting in differential changes in protein tyrosine phosphatase (PTP) α. The abnormal expression of PTPα resulted in the aberrant tyrosine phosphorylation and thus function of glycogen synthase kinase-3 (GSK-3) and protein phosphatase 2A (PP2A), causing a disruption in the phosphorylation balance of tau protein. Our data provide a novel mechanism for the epigenetic regulation of tau phosphorylation in AD, which may suggest new therapeutic targets.

## Results

### ERα and ERβ differentially regulated tau phosphorylation

**Fig 1:**
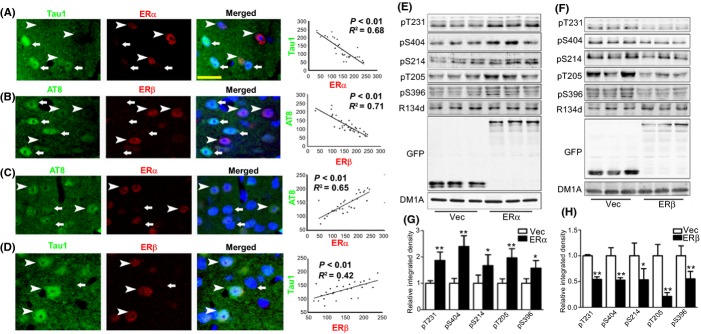
ERα overexpression and ERβ overexpression differentially regulate tau phosphorylation level. (A–D) Tg2576 mice aged 18 months were sacrificed for immunofluorescence assays with the phosphorylated tau antibody AT8 or nonphosphorylated tau antibody Tau1 and ERα or ERβ. The arrowheads indicate the ERα- (A, C) or ERβ (B, D)-positive neurons, while arrows indicate the ERα- (A, C) or ERβ (B, D)-negative neurons. The ImageJ software was used to analyze the immunofluorescence intensity, and the SigmaPlot was used for correlation analysis. (A) Tau1 and ERα, (B) AT8 and ERβ, (C) AT8 and ERα, and (D) Tau1 and ERβ. *N* = 29–34 cells from 3 individual experiments (a total of 5 mice for each group were used). Bar = 20 μm. (E–H) HEK293/tau cells were transfected with ERα or ERβ plasmids, and the samples were collected at 48 h later for Western blot. (E, F) The representative blots of pT231, pS404, pS214, pT205, and pS396 upon ERα overexpression (the GFP bands for fused protein detection) (E) and the quantitative analysis (F). (G, H) The representative blots of pT231, pS404, pS214, pT205, and pS396 upon ERβ overexpression (H) and the quantitative analysis (H). *P <* *0.05, **P <* *0.01, vs. vector transfection group. *N* = 4.

To explore the potential roles of ERα and ERβ in tau phosphorylation, we first examined the relevance of ERα and ERβ in tau phosphorylation in the prefrontal cortex of 18-month-old Tg2576 mice (pathogenic stage), which is a widely used AD mouse model. By analyzing fluorescence intensity, we found that ERα was negatively correlated with Tau1, which is a nonphosphorylated tau located at Ser198/199/202 sites, while the intensity of ERβ was negatively correlated with AT8, which is a phosphorylated tau at Ser202/Thr205 (Fig.1A,B). In addition, ERα was positively correlated with AT8, and ERβ was positively correlated with Tau1 (Fig.1C,D). These correlations are specific: First, neither ERα nor ERβ is correlated with total tau (Tau5) in the pathogenic stage (Fig. S1a,b). Second, the elevation of ERα and decreasing of ERβ are only been detected in the pathogenic stage (Fig. S1c) but not in the nonpathogenic stage of Tg2576 mice (3 months old) (Fig. S1c). Third, the alterations of ERs are not age-dependent because the ERα is not altered and ERβ displays a minor decrement in aged wild-type mice (Fig. S1c). Fourth, given that there were no significant changes in ERα in aged wild-type mice, we only examined the correlation of tau phosphorylation with ERβ and did not observe similar positive correlation of ERβ with Tau1 (Fig. S1d). These data suggested that ERα and ERβ might differentially regulate tau phosphorylation.

To validate the exact role of ERα and ERβ in tau phosphorylation, we overexpressed human ERα and ERβ in HEK293/tau cells to examine the phosphorylation levels of tau. The phosphorylation of tau at Thr205, Ser214, Thr231, Ser396, and Ser404 sites dramatically increased in response to ERα overexpression, while significantly decreased to ERβ overexpression (Fig.1E–H). These data suggested that ERα promoted tau phosphorylation, while ERβ delayed tau phosphorylation.

We then asked whether blocking ERα and ERβ could reverse the abnormal tau phosphorylation induced by ERα and ERβ overexpression. ICI 182,780 (ICI), which is a nonspecific ER antagonist, reduced ERα-caused tau hyperphosphorylation and precluded ERβ-dependent attenuation in tau phosphorylation (Fig. S2a–c). Meanwhile, ICI treatment alone did not alter the tau phosphorylation level (Fig. S2d,e), as well as the protein level of ERα and ERβ (Zhao *et al*., 2007; Zou *et al*., 2009).

We then applied an effective small hairpin (sh) RNA plasmid that specifically targeted mouse ERα or ERβ. We transfected the effective shRNA with its scrambled control into Neuro2A cells and examined tau phosphorylation by Western blot. shRNAs selectively decreased the protein levels of ERα or ERβ but not altered another receptor level (Fig. S3a–d). Silencing ERα reduced the phosphorylation of tau at multiple sites, while silencing ERβ facilitated the phosphorylation of tau at multiple sites (Fig. S3a–d). Together, these findings demonstrated that ERα and ERβ differentially regulated tau phosphorylation.

As abnormally phosphorylated tau usually aggregates to form paired helical filaments, which are the dominant component of neurofibrillary tangles in AD, we then examined the tau aggregation in the HEK293/tau cells upon ERα or ERβ treatment. We found that ERα overexpression significantly increased the phosphorylation of tau in the insoluble fraction but not the soluble fraction, as well as the amount of aggregated tau in the insoluble fraction (Fig. S4a–c). In cells overexpressed with ERβ, we detected much weaker immunoreactivities on tau phosphorylation in the soluble fraction and no signals (Fig. S4a–c) in the insoluble fraction. Those data further confirmed the critical roles of ERs in the tauopathy in AD.

### ERα and ERβ differentially regulated the tyrosine phosphorylation of GSK-3β and PP2A by PTPα

**Fig 2:**
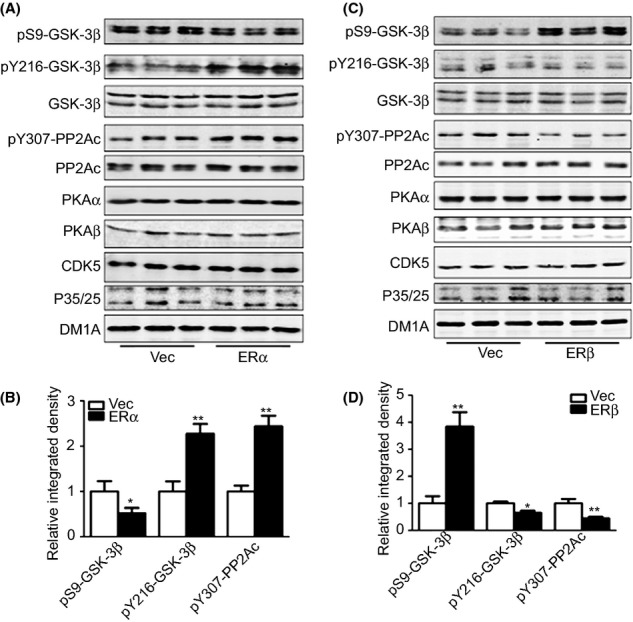
ERα overexpression and ERβ overexpression differentially regulate tau phosphorylation-related kinases and phosphatases. HEK293/tau cells were transfected with ERα or ERβ plasmids, and the samples were collected 48 h later for Western blot. (A, B) Representative blots of pS9-GSK-3β, pY216-GSK-3β, GSK-3β, pY307-PP2Ac, PP2Ac, PKAα, PKAβ, cdk5, and p35/25 upon ERα overexpression (A) and the quantitative analysis (B). (C, D) Representative blots of pS9-GSK-3β, pY216-GSK-3β, GSK-3β, pY307-PP2Ac, PP2Ac, PKAα, PKAβ, cdk5, and p35/25 upon ERβ overexpression (C) and the quantitative analysis (D). *P <* *0.05, **P <* *0.01, vs. vector transfection group. *N* = 4.

To further understand the mechanisms underlying ER-regulated tau phosphorylation, we screened the main kinases and phosphatases that are involved in tau phosphorylation. The phosphorylation of Ser9 in GSK-3β was significantly decreased, while the phosphorylation of Tyr216 in GSK-3 and Tyr307 in PP2A was dramatically increased with ERα overexpression. In the cells overexpressing ERβ, an obvious increment in the phosphorylation of Ser9 in GSK-3β and a decrement in the phosphorylation of Tyr216 in GSK-3 and Tyr307 in PP2A were observed. In addition, no alterations were found in the total levels of GSK-3β, PP2A, protein kinase (PK) Aα, PKAβ, cdk5, and p35/25 (Fig.2A–D). Treatment of the cells with ICI reversed the ERα-induced tyrosine hyperphosphorylation and restored the ERβ-induced tyrosine hypophosphorylation (Fig. S5a–c). As predicted, ICI alone did not induce the alterations of GSK-3 (Fig. S5f–g). The above results suggested abnormal and differential regulation in tyrosine phosphorylation in response to ERα and ERβ overexpression.

**Fig 3:**
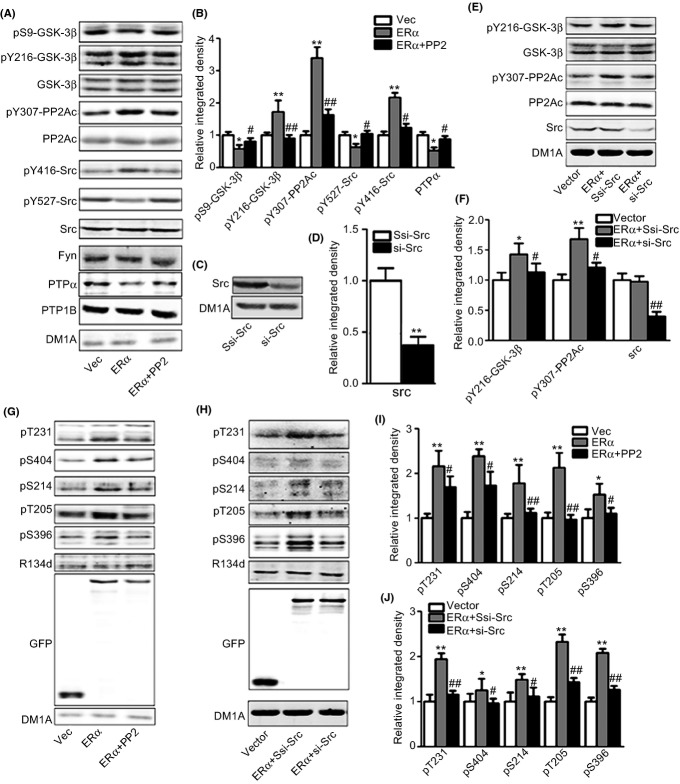
Inhibition of Src kinase reverses ERα overexpression-induced tau hyperphosphorylation. (A, B) HEK293/tau cells were transfected with ERα with or without 5 μm PP2 for 1 h, and the samples were collected for Western blot. The representative blots for pS9-GSK-3β, pY216-GSK-3β, GSK-3β, pY307-PP2Ac, PP2Ac, pY416-Src, pY527-Src, Src, Fyn, PTPα, and PTP1B are shown in (A) and the quantitative analysis is presented in (B). *P <* *0.05, **P <* *0.01, vs. vector transfection group. ^#^*P *<* *0.05, ^##^*P *<* *0.01, vs. ERα overexpression group (*N* = 3). (C, D) HEK293/tau cells were transfected with si-Src oligonucleotide (si-Src) or its scrambled control (ssi-Src), and the samples were subjected to Src antibody for Western blot (C) and quantitative analysis (D) (*N* = 3). (E, F) HEK293/tau cells were transfected with ERα plus ssi-Src (ERα+Ssi-Src) or ERα plus si-Src (ERα+si-Src) or the vectors (vector). Cell lysates were used for Western blot to detect the level of pY216-GSK-3β, GSK-3β, pY307-PP2Ac, PP2Ac, and Src (E), and the quantitative analysis was performed (F). *P <* *0.05, **P <* *0.01, vs. vector transfection group. ^#^*P *<* *0.05, ^##^*P *<* *0.01, vs. ERα overexpression group (*N* = 3). (G–J) HEK293/tau cells were transfected with ERα with or without 5 μm PP2 for 1 h (G) or with ERα plus ssi-Src (ERα) or ERα plus si-Src or the vectors (H), and the samples were subjected for the detection of tau phosphorylation. Quantitative analysis was performed in (I) and (J). *P <* *0.05, **P <* *0.01, vs. vector transfection group. ^#^*P *<* *0.05, ^##^*P *<* *0.01, vs. ERα overexpression group (*N* = 3).

As reportedly previously, tyrosine phosphorylation is mostly mediated by tyrosine kinases and phosphatases. Among those kinases and phosphatases, Src, fyn, PTPα, and PTP1B have been implicated in the pathogenesis of AD or tauopathy. We then examined the levels of those enzymes. In response to ERα overexpression, the levels of total Src, fyn, and PTP1B were not changed and the Tyr416 of Src was increased, but the Tyr527 of Src and the level of PTPα were decreased, suggesting the activation of Src and the inhibition of PTPα (Fig.3A,B). We then applied PP2, a specific Src inhibitor, and a Src shRNA (Fig.3C,D, si-Src) to test whether they reversed the ERα overexpression-induced tau and tyrosine hyperphosphorylation. Both PP2 and si-Src treatment indeed rescued the tyrosine hyperphosphorylation of GSK-3β and PP2A (Fig.3A,B,E,F) and ERα-caused phosphorylation of tau (Fig.3G–J). These data suggested that ERα facilitated tau phosphorylation by activation of GSK-3β and inactivation of PP2A through Src activation *via* PTPα inhibition.

**Fig 4:**
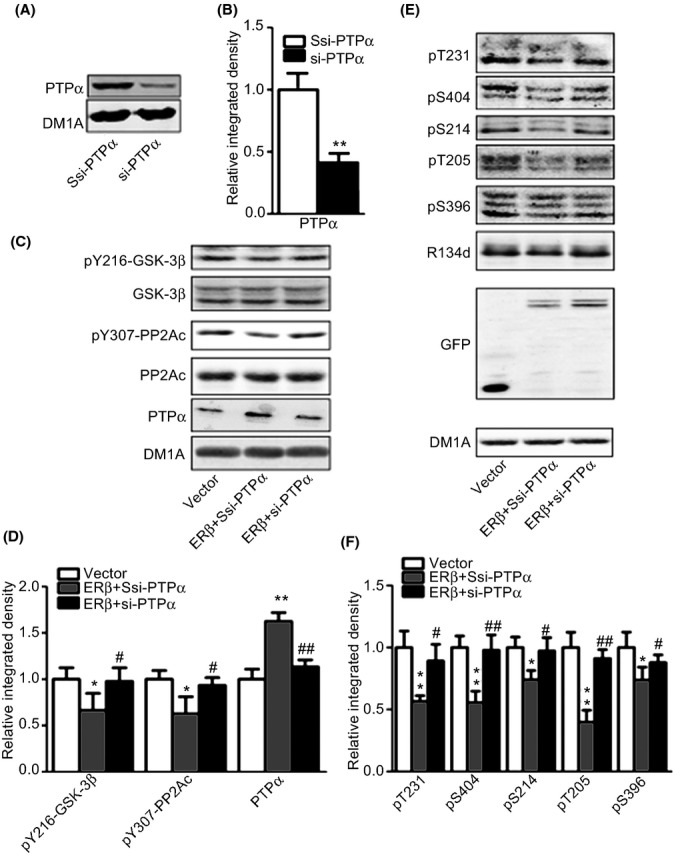
Silencing PTPα antagonizes the attenuation of tau phosphorylation by ERβ. (A, B) HEK293/tau cells were transfected with si-PTPα oligonucleotide (si-PTPα) or its scrambled control (ssi-PTPα), and the samples were probed for PTPα (A, B) (*N* = 3). (C, D) HEK293/tau cells were transfected with ERβ plus ssi-PTPα (ERβ+Ssi-PTPα) or ERβ plus si-PTPα (ERβ+si-PTPα) or the vectors (vector). The samples were used for Western blot to detect the level of pY216-GSK-3β, GSK-3β, pY307-PP2Ac, PP2Ac, and PTPα (C), and quantitative analysis was performed (D) (*N* = 4). (E, F) HEK293/tau cells were transfected with ERβ plus ssi-PTPα (ERβ) or ERβ plus si-PTPα or the vectors (E), and the samples were subjected for the detection of tau phosphorylation. The quantitative analysis was performed (F). *P <* *0.05, **P <* *0.01, vs. vector transfection group. ^#^*P *<* *0.05, ^##^*P *<* *0.01, vs. ERβ overexpression group (*N* = 4).

In response to ERβ overexpression, the levels of total Src, fyn, and PTP1B and the phosphorylation of Src were not changed, but the level of PTPα was increased, suggesting the activation of PTPα (Fig. S5a,e). Using a PTPα-specific small interfering RNA (Fig.4A,B, si-PTPα), knocking down of PTPα effectively restored the tyrosine phosphorylation of GSK-3β and PP2A (Fig.4C,D) and inhibited the attenuation of tau phosphorylation at multiple sites by ERβ (Fig.4E–F). These data suggested that ERβ weakened tau phosphorylation through the inhibition of GSK-3β and the activation of PP2A by promoting PTPα expression.

To further verify that ERα and ERβ differentially regulate PTPα pathway, we examined the tyrosine phosphorylation of GSK-3β, PP2A, and Src and the protein level of GSK-3β, PP2A, Src, fyn, and PTPα upon the ERα or ERβ silencing in N2a cells. We found that silencing ERα caused a decrease in tyrosine phosphorylation of GSK-3β and PP2A, which was accompanied with an elevation in PTPα protein level (Fig. S6a,b), while silencing ERβ induced the increment of tyrosine phosphorylation of GSK-3β and PP2A, along with the suppression of PTPα protein level (Fig. S6c,d). Furthermore, the PTPα protein level was decreased specifically in the pathogenic Tg2576 mice, but neither in nonpathogenic mice nor in aged normal mice (Fig. S6e–f), which was consistent with ERα overexpression results. In addition, silencing PTPα alone enhanced the tyrosine phosphorylation of GSK-3β and PP2Ac (Fig. S6g–h). The above data suggest that ERα and ERβ differentially regulate the expression of PTPα, which in turn results in the differential tyrosine phosphorylation of GSK-3β and PP2A.

### ERα and ERβ differentially regulated PTPα by miR-218

**Fig 5:**
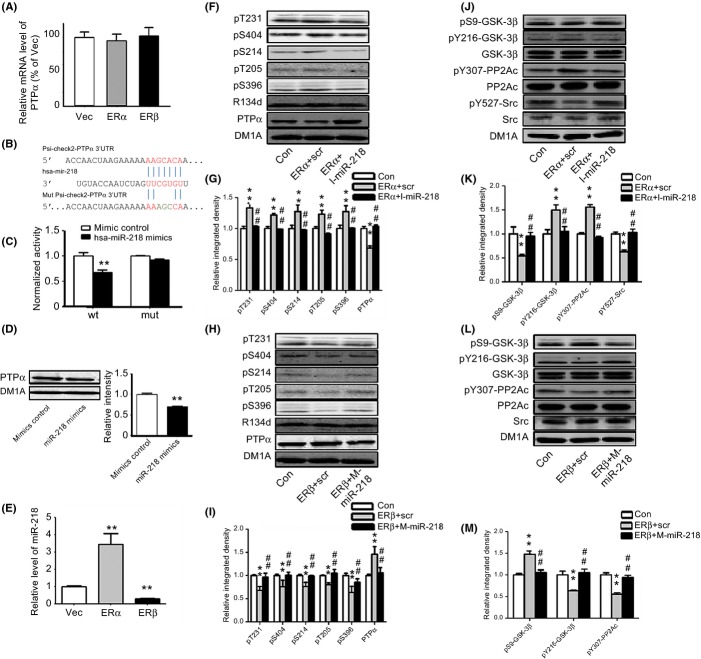
ERα overexpression and ERβ overexpression differentially regulate tau phosphorylation by miR-218. (A) HEK293/tau cells were transfected with ERα and ERβ, and the mRNA was extracted for real-time PCR examination with specific PTPα primers (*N* = 3). (B, C) The wild-type or mutant 3′-UTR of PTPα in luciferase reporter plasmids (B) were transfected with hsa-miR-218 mimics or scrambled controls in HEK293 cells for luciferase intensity detection (C). **P <* *0.01, vs. mimics control treated group (*N* = 3). (D) HEK293/tau cells were administered with hsa-miR-218 mimics, and the proteins were used for PTPα detection. (E) HEK293/tau cells were transfected with ERα and ERβ, and the miRNAs were purified as per the manufacturer’s instruction for miR-218 detection. **P <* *0.01, vs. vector transfection group (*N* = 4). (F, G) HEK293/tau cells were transfected with ERα plus hsa-miR-218 inhibitors scrambled control (ERα+scr) or ERα plus hsa-miR-218 inhibitors (ERα+I-miR-218) or the control (Con), and the samples were used for the detection of tau phosphorylation and PTPα level (F, G). **P <* *0.01, vs. control group. ^##^*P *<* *0.01, vs. ERα plus hsa-miR-218 inhibitors scrambled control treated group (*N* = 3). (H, I) HEK293/tau cells were transfected with ERβ plus hsa-miR-218 mimics control (ERβ+scr) or ERβ plus hsa-miR-218 mimics (ERβ+M-miR-218) or the control (Con), and the samples were used for examination of tau phosphorylation and PTPα (H, I). **P <* *0.01, vs. control group. ^##^*P *<* *0.01, vs. ERβ plus hsa-miR-218 mimics control treated group (*N* = 4). (J, K) HEK293/tau cells were transfected with ERα plus hsa-miR-218 inhibitors scrambled control (ERα+scr) or ERα plus hsa-miR-218 inhibitors (ERα+I-miR-218) or the control (Con), and the samples were used for the detection of pS9-GSK-3β, pY216-GSK-3β, GSK-3β, pY307-PP2Ac, PP2Ac, pY527-Src and Src (J, K). **P <* *0.01, vs. control group. ^##^*P *<* *0.01, vs. ERα plus hsa-miR-218 inhibitors scrambled control treated group. (L, M) HEK293/tau cells were transfected with ERβ plus hsa-miR-218 mimics control (ERβ+scr) or ERβ plus hsa-miR-218 mimics (ERβ+M-miR-218) or the control (Con), and the samples were used for the detection of pS9-GSK-3β, pY216-GSK-3β, GSK-3β, pY307-PP2Ac, PP2Ac, and Src (L, M). **P <* *0.01, vs. control group. ^##^*P *<* *0.01, vs. ERβ plus hsa-miR-218 mimics control treated group.

We then examined how ERα and ERβ induced the differential expression of PTPα. We first examined the mRNA levels of PTPα in HEK293/tau cells overexpressing ERα or ERβ and did not find any differences among the three groups (Fig.5A), indicating that a posttranscriptional modification might be involved in the regulation of PTPα protein levels by ERs. Because microRNAs (miRNAs) are the major regulators of posttranscriptional modification, we then performed a bioinformatics prediction with the online tool Targetscan. miR-218 had a highly conserved site that bound to the 3′ untranslated region (UTR) of *PTPRA*, the gene for PTPα (Fig.5B). To determine whether this site was targeted by miR-218, we constructed a luciferase reporter with wild-type and mutant 3′UTR segments. The wild-type reporter showed apparent inhibition while the mutant one did not when coexpressed with miR-218 (Fig.5B,C). Further, overexpression of hsa-miR-218 mimics suppression in protein levels of PTPα (Fig.5D), suggesting that miR-218 directly targeted *PTPRA*. Real-time polymerase chain reaction was then performed to determine the levels of hsa-miR-218 in ERα- and ERβ-overexpressing cells. The levels of miR-218 were increased in ERα-overexpressing cells, while the levels were decreased in ERβ-treated cells (Fig.5E). The application of anti-miR-218 restored and miR-218 mimics suppressed the levels of PTPα resulting from ERα or ERβ overexpression (Fig.5F–I), suggesting that ERα and ERβ differentially regulated PTPα through miR-218.

Finally, we tested whether correcting the miR-218 disturbances affected the tau phosphorylation levels via PTPα signals. We found that administration of anti-miR-218 to the ERα-overexpressing cells attenuated tau hyperphosphorylation, while the administration of miR-218 mimicked the effect (Fig.5F–I). Meanwhile, the anti-miR-218 application reduced the tyrosine hyperphosphorylation caused by ERα overexpression, while the miR-218 mimics restored the tyrosine hypophosphorylation induced by ERβ overexpression (Fig.5J–M). In addition, miR-218 mimics treated alone enhanced the tau phosphorylation and tyrosine phosphorylation of GSK-3 and PP2Ac, while miR-218 inhibitor treated alone suppressed the tau phosphorylation and tyrosine phosphorylation of GSK-3 and PP2Ac (Fig. S7a–f). These data further demonstrated that ERα and ERβ differentially regulated tau phosphorylation through the miR-218/PTPα pathway.

## Discussion

The accumulation of Aβ plaques and tau NFT are the two major pathological hallmarks in AD, which is the dominant form of dementia in aged people. The occurrence of AD and the global changes in AD pathology are known to significantly correlate with the loss of estrogen in women after menopause (Barnes *et al*., 2005). In a study of more than 5000 brain samples, females had more affected brain regions with NFT formations than males (Corder *et al*., 2004). Many studies have indicated that neurons are more susceptible to age-related neurodegenerative processes with declining levels of estrogen in the brain, suggesting the potential protective roles of estrogen against AD.

Estrogen exerts its neuroprotective effects through various ERs, which consist mainly of the two isoforms, ERα and ERβ. Both of these are enriched in the neocortex and hippocampus, which are two brain areas that are highly involved in AD. Many studies have described the potential roles of ERα and ERβ in AD pathogenesis. For example, in 2-month-old ERβ-knockout mice, β-amyloid deposits and apolipoprotein E are widely distributed in the brain (Zhang *et al*., 2004), suggesting that ERβ signaling disruption results in Aβ deposition. In W4 cells, estrogen treatment reduces Aβ-induced cell death through ERα-dependent pathways (Kim *et al*., 2001). These lines of data strongly suggest the critical roles of ERα and ERβ in Aβ generation. In addition, ER signaling disruption has been implicated in tauopathy. Clinical studies have suggested that there is a positive correlation between tau expression in breast cancer cells and ER expression and that this is influenced by ER signaling (Andre *et al*., 2007; Pentheroudakis *et al*., 2009). Administering ICI 182,780 does not change tau phosphorylation but reverses the tau hyperphosphorylation that is induced by okadaic acid, suggesting the involvement of ERs in tau phosphorylation (Zhang & Simpkins, 2010). In the current study, we first revealed that ERα and ERβ exerted adverse effects on tau phosphorylation. The overexpression of ERα caused tau hyperphosphorylation and aggregation, while the overexpression of ERβ induced tau hypophosphorylation. The opposite effects of ERs on tau phosphorylation were mainly caused by the differential regulation of miR-218/PTPα signaling, which in turn disturbed the balance of GSK-3β/PP2A. The differential role of ERα and ERβ in other biological processes has been well studied. For example, ERα activates while ERβ suppresses the gene expression of cyclin D1 (Liu *et al*., 2002). ERα mediates the cancer-promoting effects of estrogens, and ERβ inhibits the proliferation of breast cancer cells by repressing c-myc and cyclin A gene transcription (Paruthiyil *et al*., 2004). In HC11 mammary epithelial cells, ERα drives proliferation in response to E2, while ERβ is growth inhibitory (Helguero *et al*., 2005). In the nervous system, ERα could impair memories for socially acquired food preferences, while ERβ could enhance the acquisition of the task (Clipperton *et al*., 2008). Our data suggested the different roles of ERα and ERβ in AD. ERα may have deleterious effects, but ERβ may have protective effects.

As one of the most important noncoding RNAs, the roles of miRNA in AD pathogenesis have been well studied. After the first miRNA array report in AD appeared in 2007 (Lukiw, 2007), multiple studies have identified a number of disrupted miRNAs in AD. It has been reported that the decrement in miR-29a/b-1 results in the increased expression of β-secretase 1, which is the most important β-secretase, and the overproduction of Aβ (Hebert *et al*., 2008). The depletion of Dicer, which is a RNase that is required for miRNA maturation, induces the hyperphosphorylation of tau, indicating the critical roles of miRNAs in tauopathy (Hebert *et al*., 2010). In progressive supranuclear palsy, the loss of miR-132 is associated with tau exon10 inclusion, which further induces an imbalance of the 4R/3R-Tau ratio in neuronal cells (Smith *et al*., 2011). In addition, two members of the miR-16 family, miR-15a and miR-15b, are downregulated in AD brain and cerebrospinal fluid, respectively (Cogswell *et al*., 2008). Both of these have been suggested to target the 3′UTR of extracellular signal-regulated kinase 1 (Hebert *et al*., 2012), which is an important kinase for tau phosphorylation in AD (Ferrer *et al*., 2001). These lines of data provide the preliminary links between tau phosphorylation and miRNA dysfunction, but direct experimental evidence is missing. Here, we found that miR-218 acted as the axis in regulating tau phosphorylation upon ERα or ERβ activation. Specifically, ERα overexpression increased miR-218 expression and tau phosphorylation, and suppression of the increased miR-218 levels rescued the tau hyperphosphorylation that was caused by ERα. ERβ overexpression decreased miR-218 expression and tau phosphorylation, and supplementation of miR-218 mimics blocked the alleviation of the tau phosphorylation that was induced by ERβ. As previously reported, miR-218 accumulates in the hippocampus (Bak *et al*., 2008) and is activated during neuronal differentiation (Sempere *et al*., 2004). A number of miR-218 targets have been identified to exert diverse functions in the brain. For example, miR-218 targets multiple components of receptor tyrosine kinase signaling pathways, and miR-218 repression increases the abundance and activity of multiple receptor tyrosine kinase effectors (Mathew *et al*., 2014). In our study, miR-218 specifically targeted the 3′UTR of *PTPA*, the gene for PTPα, and regulated the tyrosine phosphorylation of GSK-3β and PP2A. Our study thus extended the potential role of miR-218 in the brain.

PTPα belongs to the protein tyrosine phosphatase family that regulates a variety of cellular processes, including cell growth, differentiation, and the mitotic cycle (Pallen, 2003). The expression of PTPα is accompanied by Src dephosphorylation and activation in the developmental stage of neurons (den Hertog *et al*., 1993), and the stable expression of PTPα will activate Src and mediate epidermal growth factor-induced neurite outgrowth (Yang *et al*., 2002). Moreover, PTPα combines with the neural cell adhesion molecule contactin to form a receptor complex that plays an important role in neuronal cell interactions (Berglund *et al*., 1999) and in hippocampal synaptic plasticity (Murai *et al*., 2002). Most importantly, PTPα has been implicated in tyrosine phosphorylation and in the regulation of the activity of its substrates. For example, the physical interactions of phosphoinositide 3-kinase and protein kinase Cδ with PTPα play a role in the activation of mitogen-activated protein kinase (Stetak *et al*., 2001). Here, we first reported that the phosphorylation of tyrosine 216 in GSK-3β and tyrosine 307 in PP2A was regulated by PTPα and concordant with the alterations in GSK-3β and PP2A activity, which in turn resulted in abnormal tau phosphorylation levels. The activation of PTPα increased the dephosphorylation of tyrosine sites and induced the inhibition of GSK-3β and the activation of PP2A, which further suppressed the phosphorylation of tau protein. Although PTPα changes in AD have not been studied, the attenuation of tau phosphorylation by restoring PTPα levels is a potential therapeutic strategy.

Taken together, our study demonstrated the differential regulation of ERα and ERβ on tau phosphorylation through miR-218/PTPα signals for the first time and provided data on the fundamental role of the miR-218/PTPα pathway in tauopathy.

## Experimental procedures

### Antibodies and reagents

All the primary antibodies used in this study are list in Table1.

**Table 1:** Primary antibodies used in the current study

Antibodies	Type	WB dilution	IHC dilution	References and sources
Tau1	mAb	–	1:200	Millipore (Billerica, MA, USA)
AT8	mAb	–	1:100	Thermo (Waltham, MA USA)
ERα	pAb^a^	1:1000	1:100	Millipore
ERβ	pAb	1:500	1:50	Thermo
pS396	pAb	1:1000	–	SAB (Pearland, TX, USA)
pS404	pAb	–	–	SAB
pT231	pAb	1:1000	–	SAB
pS214	pAb	1:500	–	SAB
pT205	pAb	1:500	–	SAB
R134d	pAb	1:500	–	Gift from Dr. Khalid Iqbal*
GFP	pAb	1:1000	–	Abcam (Cambridge, UK)
DM1A	mAb	1:1000	–	Sigma (St. Louis, MO, USA)
GSK-3β	pAb	1:1000	–	SAB
pS9-GSK-3β	pAb	1:1000	–	Cell Signaling (Danvers, MA, USA)
pY216-GSK-3β	mAb	1:1000	–	Millipore
PP2Ac	mAb	1:1000	–	Millipore
pY307-PP2Ac	pAb	1:1000	–	Abcam
CDK5	mAb	1:1000	–	Santa Cruz Biotechnology (Dallas, Texas USA)
P35/25	pAb	1:1000	–	Santa Cruz Biotechnology (Dallas, Texas USA)
PKAα	pAb	1:1000	–	Santa Cruz Biotechnology (Dallas, Texas USA)
PKAβ	pAb	1:1000	–	Santa Cruz Biotechnology (Dallas, Texas USA)
pY416-Src	pAb	1:1000	–	Cell Signaling
pY527-Src	pAb	1:1000	–	Sigma
Src	mAb	1:1000	–	Millipore
Fyn	pAb	1:1000	–	Cell Signaling
PTPα	pAb	1:1000	–	Upstate
PTP1B	pAb	1:1000	–	Abcam
Goat anti-mouse peroxidase	–	1:5000	–	Pierce Chemical Company
Goat anti-rabbit peroxidase	–	1:5000	–	Pierce Chemical Company

* Rabbit polyclonal antibody R134d against total tau was a gift from Drs. K. Iqbal and I. Grundke-Iqbal (New York State Institute for Basic Research, Staten Island, NY, USA).

ICI 182,780 was purchased from Tocris Bioscience (Bristol, UK) and dissolved in DMSO to 100 μm as stocking solution. Specific inhibitor PP2 was purchased from Merck KGaA (Darmstadt, Germany) and dissolved in DMSO to 5 mm for stock. Lipofectamine 2000 was purchased from Invitrogen (San Diego, CA, USA). Cell culture media were from Gibco (San Diego, CA, USA). Plasmids containing the human ERα and ERβ cDNAs were constructed according to the following sequences: NM_001122741 and NM_001437 to pEGFP-N1. shRNA-ERβ plasmid toward mouse ERβ (NM_207707.1) was constructed to vector GV102 by Neuron Biotech Inc. (Shanghai, China). si-ERα oligonucleotide toward mouse was synthesized using the sequence published before (Carbonaro *et al*., 2009). Si-Src and si-PTPα oligonucleotides toward human were synthesized according to previous publications (Zheng *et al*., 2008). The primers used for PTPα detection are as follows: forward, 5′-AGTGGTCTGATATGTGTCAGTGC-3′; reverse, 5′-GGTTCTGCCGTTGATGAGTTA-3′. The primers for hsa-miR-218 detection, the hsa-miR-218 mimics (Cat. No. miR10000275-1-2) and inhibitors (Cat. No. miR20000275-1-2), as well as their scrambled controls, were purchased from Ribobio Co., Ltd (Guangzhou, China).

### Animals and treatment

Eighteen-month-old male Tg2576 mice were purchased from the Jackson Laboratory (Bar Harbor, ME, USA) and housed in a room on a 12:12 hr light–dark cycle and 22 ± 2 °C with water and food *ad libitum* for at least 2 weeks before the day of experimentation. All animal experiments were performed according to the ‘Policies on the Use of Animals and Humans in Neuroscience Research’ revised and approved by the Society for Neuroscience in 1995.

### Cell culture and treatments

HEK293/tau cells (HEK293 cells stably transfected with the longest human tau (tau441) cDNA) were cultured in DMEM in the presence of 200 μg mL^−1^ G418 with 10% fetal bovine serum (FBS, vol/vol), and mouse neuroblastoma 2a (N2a) cells (kindly gift by Dr. Huaxi Xu at Xiamen University) were seeded in six-well plates in DMEM with 10% fetal bovine serum (FBS, vol/vol). Both cells were cultured in a humidified atmosphere of 5% CO_2_ at 37 °C. The cells were cultured for at least 24 h after plating, and when grown to 80–90% confluence, the culture medium was replaced with serum- and antibiotic-free DMEM prior to treatment.

Plasmids used for transfection were amplified and purified by Qiagen kit (Qiagen, Hilden, Germany) according to the manufacturer’s instruction. Briefly, HEK293/tau or N2a cells were seeded in six-well plates, grown to 60–70% confluence, and then cultured in serum- and antibiotic-free OPTI-MEM for 4 h. Plasmids were transfected with Lipofectamine 2000 (Invitrogen) according to the manufacturer’s instruction. Cells transfected with GFP constructs were visualized at 48 h after transfection by an Olympus IX70 microscope with a 209LCPlanF1 lens (Olympus Corporation, Matsue, Shimane Japan). For double transfection, the plasmid and oligonucleotides were added to OPTI-MEM, respectively, at the last step of transfection. 48–60 h after transfection, cells were treated with 100 nM ICI 182,780 for 1 h or 5 μm PP2 for 1 h. Then, the media were removed and the cells were harvested and stored at −20 °C for further experiments.

### Immunofluorescence and confocal microscopy

A total of 5 mice for each group were sacrificed by overdose chloral hydrate (1 g kg^−1^) and perfused through aorta with 100 mL 0.9% NaCl followed by 400 mL phosphate buffer containing 4% paraformaldehyde. About 2 h later, brains were removed and postfixed in perfusate overnight and then cut into sections (15–20 μm) with vibratome (Leica, Nussloch, Germany; S100, TPI). The sections of mice brain were collected consecutively in PBS for immunofluorescence staining. Free-floating sections were incubated with bovine serum albumin (BSA) to block nonspecific sites for 30 min at 25 °C. Sections were then incubated overnight at 4 °C with primary antibodies rabbit polyclonal ERα or ERβ antibody for 48 h, and after washing with PBS, sections were subsequently incubated with mouse monoclonal Tau1, Tau5, or AT8 for 48 h. After washed with PBS for 30 min, sections were subsequently incubated secondary antibodies Alexa Fluor 488 (donkey anti-mouse) or Alexa Fluor 546 (goat anti-rabbit) for 1 h at 37 °C. The prefrontal cortex region was chosen for imaging using a laser confocal microscope (LSM710 Carl Zeiss, München, Germany) (Chen *et al*., 2012).

### Real-time PCR

The total RNA from the cells was extracted by TRIzol reagent (Invitrogen), and 1 μg RNA was reversely transcripted. qRT–PCR was performed on ABI StepOne Plus using SYBR Green ® Premix Ex Taq (Takara, Tokyo, Japan). MicroRNA was extracted using miRNA isolation kit (Tiangen, Beijing, China). Reactions were prepared in a total volume of 10 μL containing 0.5 μL cDNA (100 ng μL^−1^), 1 μL of each 2 μm primer (300 mm each), 5 μL of SYBR Green, and 2.5 μL RNase/DNase-free sterile water. Blank controls were run in triplicate for each master mix. The cycle conditions were set as follows: initial template denaturation at 95 °C for 1 min, followed by 40 cycles of denaturation at 95 °C for 5 s, and combined primer annealing/at 60 °C for 30 s, and elongation at 72 °C for 30 s. This cycle was followed by a melting curve analysis, ranging from 60 to 95 °C, with temperature increasing by steps of 0.5 °C every 10 s.

### Western blotting

Cells were rinsed twice in phosphate-buffered saline at pH 7.5 and lysed with buffer containing 50 mm Tris-HCl, pH 8.0, 150 mm NaCl, 1% NP-40, 0.5% sodium deoxycholate, 0.1% SDS, 0.02% NaN3, 100 μg mL^−1^ PMSF, and 10 μg mL^−1^ each of the protease inhibitors (leupeptin, aprotinin, and pepstatin A) followed by boiling for 5–6 min, and then sonicated for 5 s on ice. The cell lysates were then centrifuged at 12 000 *g* for 5 min at 4 °C; aliquots of supernatants were added to one-third volume of 4× sample buffer, 10% beta-mercaptoethanol (ME), and 0.05% bromophenol blue and then stored at −20 °C or used immediately. Protein concentration was quantitated using the BCA Protein Assay Reagent Kit (Pierce, Rockford, IL, USA) (Jiang *et al*., 2011; Liu *et al*., 2015).

Equal amounts of protein were separated by SDS–polyacrylamide gel electrophoresis (10% gel) and transferred to nitrocellulose membrane. The membranes were blocked with 5% nonfat milk dissolved in PBS (50 mm Tris-HCl, pH 7.6, 150 mm NaCl) for 30 min–1 h and probed with primary antibodies overnight at 4 °C. Then, the blots were incubated with goat anti-mouse or anti-rabbit conjugated to IRDye 800 (Rockland Immunochemicals) (1:15000) for 1 h at 25 °C. The protein bands were visualized and quantified by the Odyssey infrared imaging system (LI-COR, Lincoln, Nebraska, USA). The levels of the phosphorylated protein tau, PP2Ac, GSK-3β, and Src were normalized against the total protein tau, PP2Ac, GSK-3β, Src, and PTPα. The amount of protein was expressed as relative level of the sum optical density against controls.

For tau aggregation analysis (Ishihara *et al*., 1999; Li *et al*., 2014), the cells were homogenized in cold RAB Hi-Salt buffer (0.1 m MES pH 7.0, 1 mm EGTA, 0.5 mm MgSO4, 0.75 m NaCl, 0.1 m EDTA) containing protease inhibitors (100 μg mL^−1^ PMSF) and centrifuged at 50 000 *g* for 40 min in 4 °C, and the supernatants were saved as the RAB-soluble fraction. The RAB-insoluble pellets were sonicated in sample buffer containing 0.2 g mL^−1^ sucrose, 18.5 mm Tris (pH 6.8), 2 mm EDTA, 80 mm DTT, and 2% SDS and centrifuged at 50 000 *g* for 20 min in 4 °C. The supernatant was discarded, and the pellet was homogenized in cold RIPA buffer (50 mm Tris pH 7.4, 150 mm NaCl, 1% NP-40, 0.5% sodium deoxycholate, 0.1% SDS, 1 mm EDTA, 50 mm natrium fluoride) with protease inhibitor and centrifuged at 50 000 *g* for 20 min in 4 °C. The supernatant was saved as RIPA-soluble fraction, and the pellet was extracted in 70% formic acid as FA fraction. Fractions were analyzed by SDS-PAGE.

### Statistical analysis

Data were expressed as mean ± SD and analyzed using spss 10.0 statistical software (SPSS Inc., Chicago, IL, USA). The one-way anova procedure followed by LSD’s *post hoc* tests was used to determine the different means among groups.

## Funding

This work was supported in part by the National Natural Science Foundation of China (81361120245, 31201011, 81261120570, 91232302, 81428009), MOST International Collaboration Grant (2011DFG33250), and Program for Changjiang Scholars and Innovative Research Team in University (IRT13016).

## Author contributions

L.Q.Z. initiated, designed, and directed this study. Y.S.X., F.F.L., D.L., H.Z.H., N.W., and L.T. performed the experiments. J.G.C. and J.Z.W. provided partial financial support. C.X.G., Y.L., and J.Z.W. provided some comments. Y.S.X., F.F.L., H.Y.M, and L.Q.Z. wrote the manuscript. All authors read and approved the final manuscript.

## Conflict of interest

All the authors declare that they have no conflict of interest in relation to this study.

## Abbreviations

ER: estrogen receptor
AD: Alzheimer’s disease
PTPα: protein tyrosine phosphatase α
NFTs: neurofibrillary tangles
GSK-3: glycogen synthase kinase-3
PP2A: protein phosphatase 2A
ICI: ICI 182,780
Aβ: β-amyloid protein

## Supporting Information

**Fig. S1.** The correlation of ERs with tau phosphorylation are specific. **Fig. S2.** ICI 182,780 reverses tau phosphorylation induced by ERα and ERβ overexpression. **Fig. S3.** Silencing of ERα or ERβ differentially regulates tau phosphorylation. **Fig. S4.** Overexpression of ERα but not ERβ promotes the tau aggregation. **Fig. S5.** ICI 182,780 reverses tyrosine phospohrylation of GSK-3β, PP2A, Src and the protein level of GSK-3β, PP2A, Src, PTPα induced by ERα and ERβ overexpression. **Fig. S6.** Silencing of ERα or ERβ differentially regulates PTPα signals. **Fig. S7.** The effects of miR-218 mimics and inhibitors on tau phosphorylation and tyrosine phosphorylation of GSK-3β and PP2Ac.
