# Supplementary material for: Opposite effects of two estrogen receptors on tau phosphorylation through disparate effects on the miR-218/PTPA pathway
Source: Aging Cell. 2015 Jun 26;14(5):867–77. doi: 10.1111/acel.12366 (PMC4568974; doi:10.1111/acel.12366)
Supplement: Supplementary file 1 [file acel0014-0867-sd1.docx]

**Opposite effects of two estrogen receptors on tau phosphorylation through disparate effects on the miR-218/PTPA pathway**

Yan-Si Xiong^1,2,#^, Fang-Fang Liu^1,2,#^, Dan Liu^2,3,4^, He-Zhou Huang^1,2^, Na Wei^1,2^, Lu Tan^1,2^, Jian-Guo Chen^2, 5^, Heng-Ye Man^6^, Cheng-Xin Gong^7^, Youming Lu^2^, Jian-Zhi Wang^1,2^, Ling-Qiang Zhu^1,2,4,*^


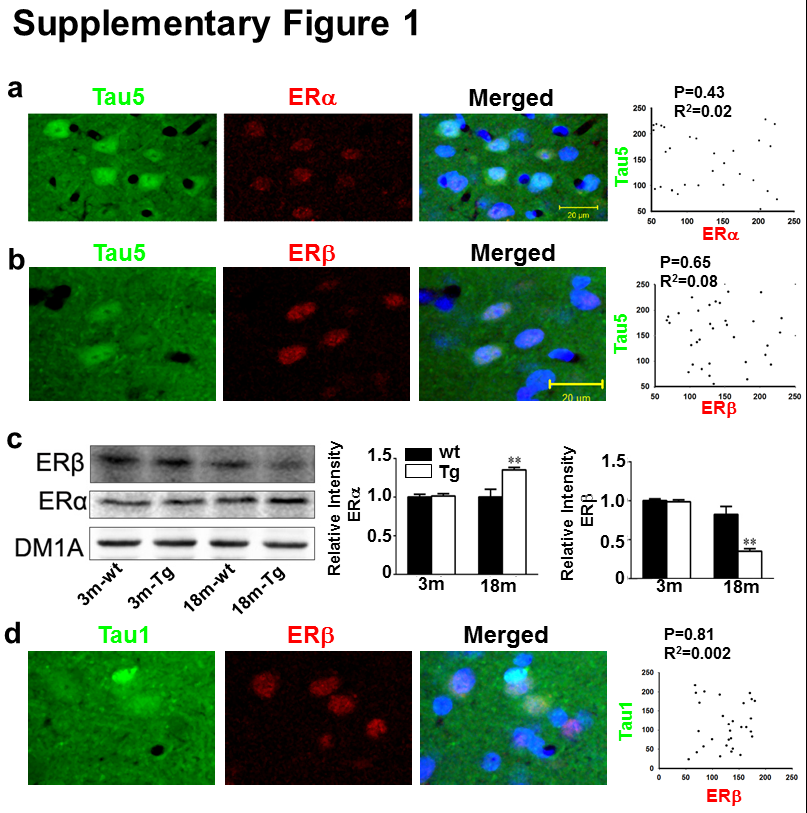


**Supplementary Figure 1. The correlation of ERs with tau phosphorylation are specific.**

(a-b) Tg2576 mice at 18-month were sacrificed for immunofluorescence assays with the total tau antibody Tau5 and ERα or ERβ. The Image J software was used to analysis the immunofluorescence intensity and the Sigmaplot was used for correlation analysis. Bar=20μm. N=4.

(c) The prefrontal cortical samples from 18-month or 3-month Tg2576 mice (Tg) and age-matched wild type littermates (wt) were homogenated and subjected for the ERα and ERβ detection by western blotting. ***P<0.01*, vs wt group. N=4.

(d) Wild C57 mice at 18-month were sacrificed for immunofluorescence assays with the Tau 1 and ERβ. The Image J software was used to analysis the immunofluorescence intensity and the Sigmaplot was used for correlation analysis. N=4.


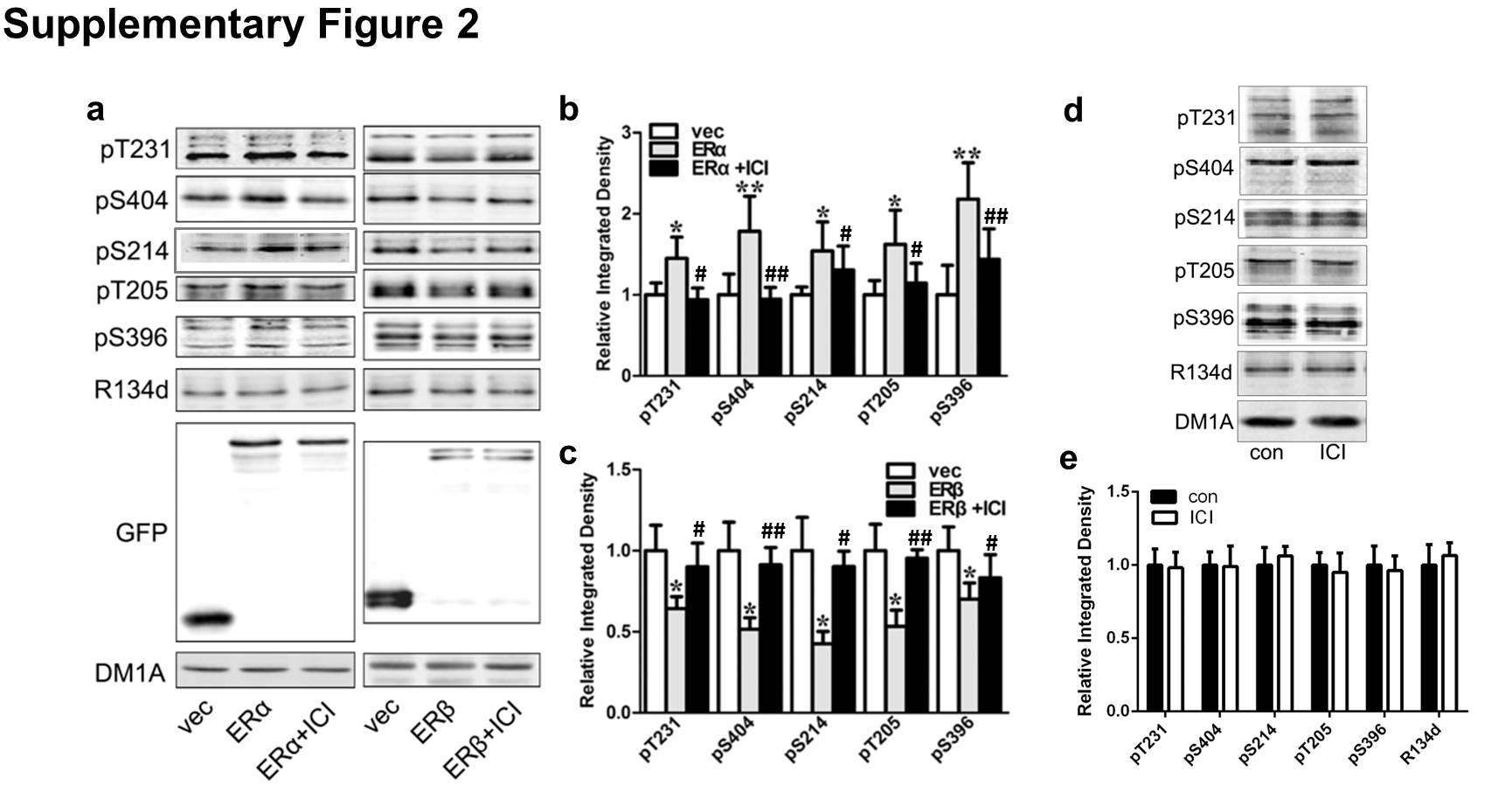


**Supplementary Figure 2. ICI 182,780 reverses tau phosphorylation induced by ERα and ERβ overexpression.**

HEK293/tau cells were transfected with ERα or ERβ plasmids and treated with ICI 182,780 100 nM for 1h at 48-60 h after transfection, the samples were collected then for western blot. (a, d) The representative blots of pT231, pS404, pS214, pT205, pS396 upon ERα or ERβ overexpression, with or without ICI 182,780 (ICI) treatment or ICI treated alone (d). (b, c, e) The quantitative analysis. **P*<0.05, ** *P*<0.01, *vs* with vector transfection group. #*P*<0.05, ## *P*<0.01, *vs* with ERα or ERβ overexpression group (N=4).


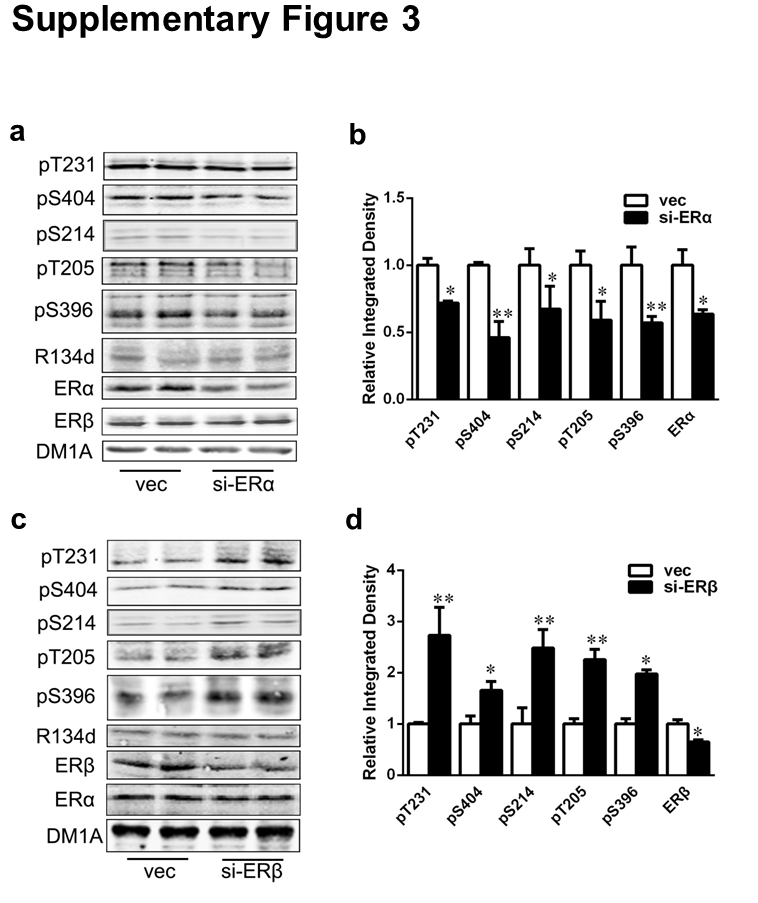


**Supplementary Figure 3. Silencing of ERα or ERβ differentially regulates tau phosphorylation.**

HEK293/tau cells were transfected with ERα or ERβ shRNA or scrambled control (vec) and the samples were collected for western blot. (a-b) The representative blots of pT231, pS404, pS214, pT205, pS396, ERα and ERβ upon ERα silencing (a) and the quantitative analysis (b) (N=4). (c-d) The representative blots of pT231, pS404, pS214, pT205, pS396, ERα and ERβ upon ERβ silencing (c) and the quantitative analysis (d). **P*<0.05, ** *P*<0.01, *vs* with scrambled control transfected group (N=4).


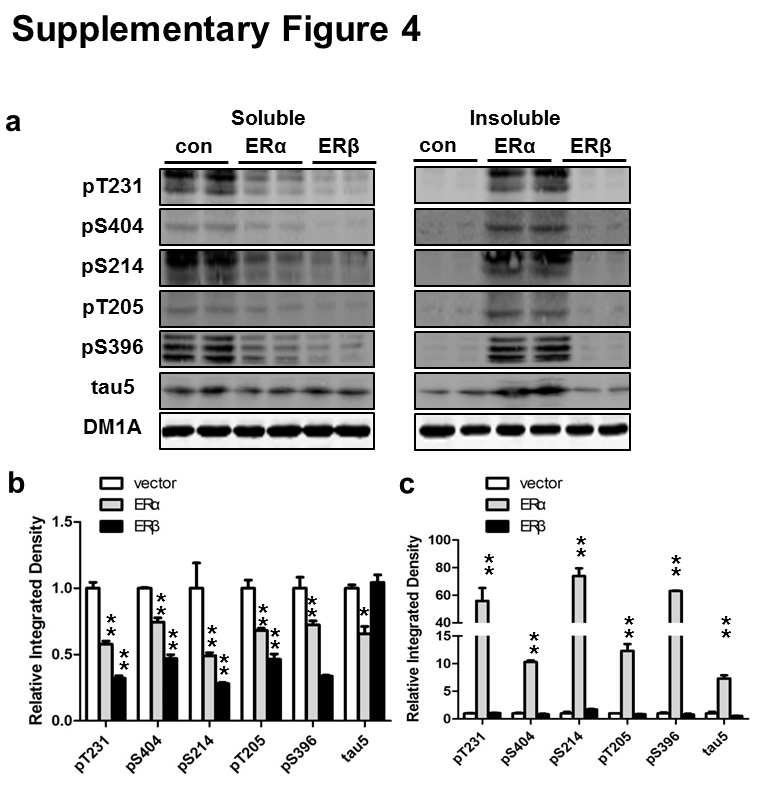


**Supplementary Figure 4. Overexpression of ERα but not ERβ promotes the tau aggregation**

HEK293/tau cells were transfected with ERα or ERβ, and the cell lysis were separated to soluable and insoluable fractions. Then, the different fractions were subjected to detect the tau phosphorylation by western blot. (a) The representative blots of pT231, pS404, pS214, pT205, pS396, Tau 5 from RIPA fraction (soluable) and 70% formic acid fraction (insoluable) upon the overexpression of ERα and ERβ. (b-c) The quantitative analysis for tau phosphorylation in soluable (b) and insoluable (c) fractions.* *P*<0.05, ** *P*<0.01, *vs* with vector transfection group. N=4


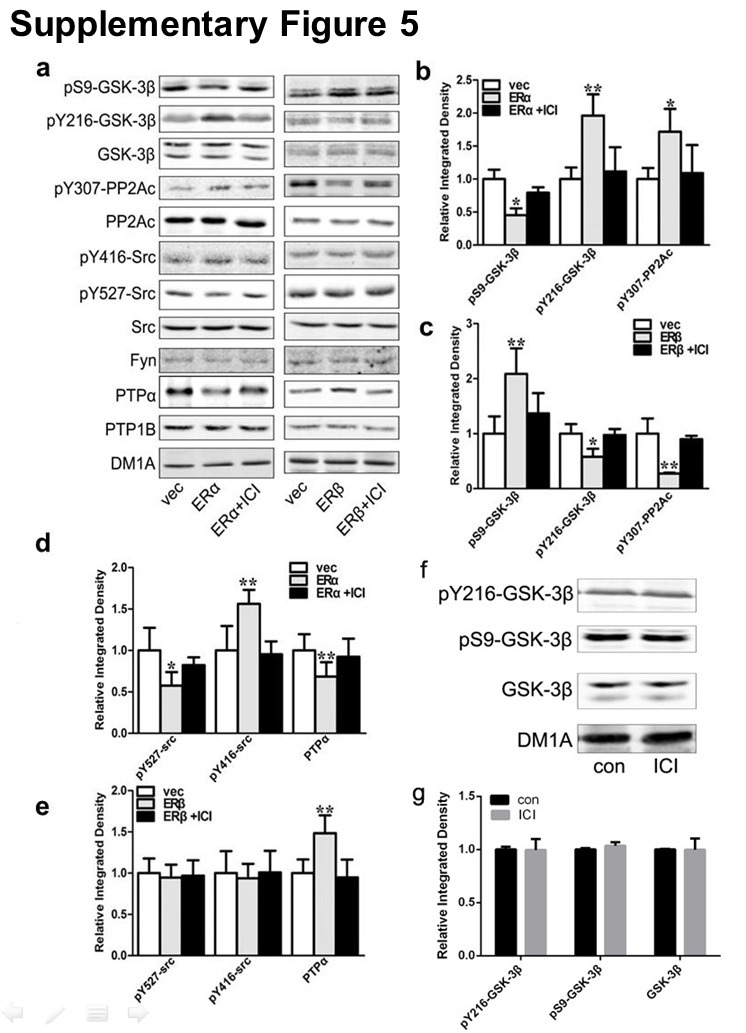


**Supplementary Figure 5: ICI 182,780 reverses tyrosine phospohrylation of GSK-3β, PP2A, Src and the protein level of GSK-3β, PP2A, Src, PTPα induced by ERα and ERβ overexpression.**

HEK293/tau cells were transfected with ERα or ERβ plasmids and treated with ICI 182,780 100 nM for 1h at 48-60 h after transfection, the samples were collected then for western blot. (a) The representative blots of pSer9-GSK-3β (pS9-GSK-3β), pTyr216-GSK-3β (pY216-GSK-3β), GSK-3β, pTyr307-PP2ac (pY307-PP2ac), pTyr416-Src (pY416-Src), pTyr527-Src (pY527-Src), Src, Fyn, PTPα, PTP1B upon ERα or ERβ overexpression, with or without ICI 182,780 (ICI) treatment. (b-e) The quantitative analysis. *P<0.05, ** P<0.01, vs with vector transfection group (N=4). (f-g) HEK293/tau cells were transfected with vector or co-treated with ICI 182,780 100 nM for 1h at 48-60 h after transfection, the lysis were collected then for western blot to determine the pSer9-GSK-3β (pS9-GSK-3β), pTyr216-GSK-3β (pY216-GSK-3β), GSK-3β level (f) and the quantitative analysis were performed.


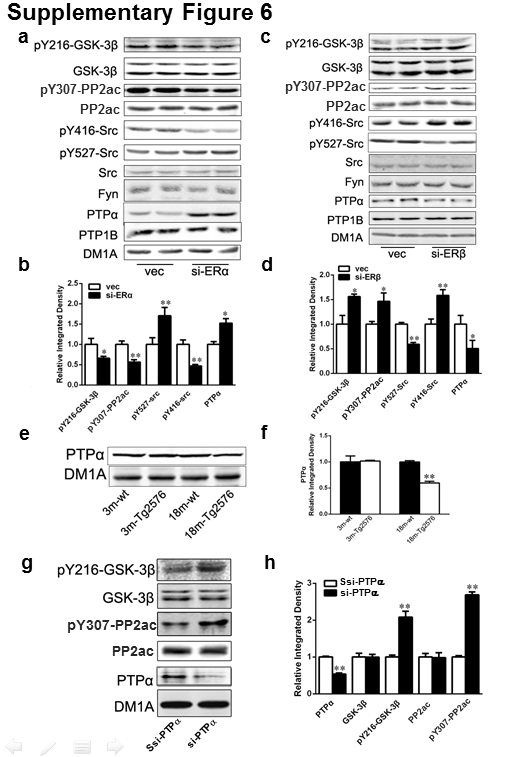


**Supplementary Figure 6. Silencing of ERα or ERβ differentially regulates PTPα signals.**

HEK293/tau cells were transfected with ERα or ERβ shRNA or scrambled control (vec) and the samples were collected for western blot. (a-b) The representative blots of pS9-GSK-3β, pY216-GSK-3β, GSK-3β, pY307-PP2Ac, PP2Ac, pY416-Src, pY527-Src, Src, Fyn, PTPα, PTP1B upon ERα silencing (a) and the quantitative analysis (b). (c-d) The representative blots of pS9-GSK-3β, pY216-GSK-3β, GSK-3β, pY307-PP2Ac, PP2Ac, pY416-Src, pY527-Src, Src, Fyn, PTPα, PTP1B upon ERβ silencing (c) and the quantitative analysis (d). **P*<0.05, ** *P*<0.01, *vs* with scrambled control transfected group.

(e-f) The alterations of PTPα level in the hippocampus of Tg2576 mice. (e) The representative blots (f) the quantitative analysis. ***P*<0.01, *vs* with wild type littermates at 3 month.

(g-h) The HEK293/tau cells were transfected with PTPα siRNA and subjected to the examination of pY216-GSK-3β, GSK-3β, pY307-PP2Ac, PP2Ac. (g) The representative blots and (h) the quantitative analysis. ** *P*<0.01, *vs* with scrambled control transfected group.


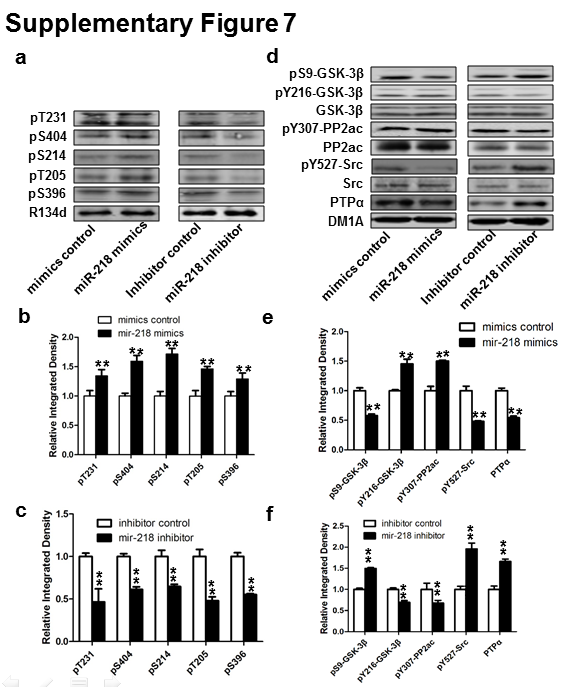


**Supplementary Figure 7. The effects of miR-218 mimics and inhibitors on tau phosphorylation and tyrosine phosphorylation of GSK-3β and PP2ac.**

(a-c) HEK293/tau cells were transfected with hsa-miR-218 mimics control (mimics control) or hsa-miR-218 mimics (miR-218 mimics) and the samples were used for tau phosphorylation level examination by western blot (a) and quantitative analysis (b-c). ** *P*<0.01, *vs* hsa-miR-218 mimics control or inhibitor control treated group.

(d-f) HEK293/tau cells were transfected with hsa-miR-218 inhibitor control (inhibitor control) or hsa-miR-218 inhibitor (miR-218 inhibitor) and the samples were used for pS9-GSK-3β, pY216-GSK-3β, GSK-3β, pY307-PP2Ac, PP2Ac, pY416-Src, Src examination by western blot (d) and quantitative analysis (e-f). ** *P*<0.01, *vs* hsa-miR-218 mimics control or inhibitor control treated group.
